# Editorial: Innovative approaches for disease modeling using organoids and organ-on-a-chip

**DOI:** 10.3389/fbioe.2026.1991263

**Published:** 2026-09-18

**Authors:** Sándor Valkai

**Affiliations:** Institute of Biophysics, HUN-REN Biological Research Centre, Szeged, Hungary

**Keywords:** bioelectronics chip, biomarker, breastcancer detection, Lab-on-a chip, multi electrode array (MEA), organ-on-a chip, patient derived organoids

We have addressed this Research Topic, ***Innovative Approaches for Disease Modeling Using Organoids and Organ-On-A-Chip***, to consolidate and clarify the current advancements in organoid and organ-on-a-chip methodologies to publish the freshest knowledge about disease mechanisms, drug responses, and the potential applications in personalized medicine. We hoped very much that these new results, reported here, help in the near future to elaborate on and design tailored therapeutic strategies.

Hereby, I enclose this Research Topic in which five articles are published. I can note proudly that two of these articles report on original research and three summarize the newest results from special areas of the contemporal research.

The first original research article is as follows: *The Integrated transcriptomic and immune-associated network analysis of breast cancer patient-derived organoids reveals candidate inflammatory biomarkers* (Scippa et al.).

In this article, the authors present their results from studies based on patient-derived organoids (PDOs). According to this scientific report, 646 differentially expressed genes were identified. In breast cancer, translational machinery and ribosome biogenesis are the dominant and statistically significant processes, which is consistent with hyperactivated protein synthesis in cancer cells. This suggests that in breast cancer PDOs, the IL1RAP and IRF2BP2 (from the STRING database) are in immune-related and may be important candidates for biomarker identification. The Authors have used integrated transcriptomic enrichment and network-based analyses for a robust strategy to discover biomarkers. This methodologically transparent framework the authors utilized has highlighted some potential targets in breast cancer.

The second original research article is as follows: *Unraveling Hydra bioelectrical activity on multielectrode array* (Blasio et al.).

This article concerns the utilization of devices for collecting experimental data, especially the Multi Electrode Array (MEA). In this research study, a millimeter-sized organism (*Hydra vulgaris*) was studied, applying a commercially available multielectrode recording system. The paper is actually not about organoids but a living creature that is the size of a regular organoid but has a more complex biological system than that. The data were comprehensively analysed by their own MATLAB-based algorithm, searching for extracellular electric signals. The authors introduced two different configurations in order to record these bioelectric patterns. They have demonstrated that *H. vulgaris* is a versatile model in bioelectronics. Their result support that *Hydra* can be used as a testing system to develop and validate new advanced microengineered devices, designed for fundamental studies on neuronal circuits and neuromodulation.

We also have three reviews. The first is *A narrative review of spatial multi-omics and organ-on-a-chip technologies for cardio-cerebral-renal crosstalk* (Cheng et al.).

In this review, the authors collect and conclude the possibilities and promises of spatial multi-omics and organ-on-a-chip (OOC) technologies. Their plan was to evaluate the efficacy and safety of spatial multi-omics and organ-on-a-chip (OOC) technologies, comparing them to conventional methods in decoding cardio-cerebral-renal crosstalk for systemic disorders including sensitivity, resolution, and detection accuracy. Based on 34 studies, they conclude that there is a novel paradigm for decoding multi-organ communication that reveal molecular mechanisms (e.g., exosomal miRNA regulation) and provide clinically actionable biomarkers.

The next review is *Cancer metastasis in vitro models–can 3D biofabrication and microfluidics sow the seeds in the right soil* (Sbirkov et al.).

This is an analysis of works that study the cancer metastasis applications of microfluidics, reinforced by 3D fabrication (bioprinting with micrometer precision and microfluidic biosensors that measure cellular parameters). They conclude that this way, tools can be provided for the creation of vascularized multi-organ experimental systems. Furthermore, it is a promising strategy to integrate physiological sensors (pH and oxygen) and agitators into the chip since the most important cell parameters can be measured directly. What is more, these moduls can be connected to each other to create in such a way models that reflect the complexity of human organs much more accurately than an organoid (made one type of cells). The result of such an integration can be a highly sophisticated ‘human avatars on a chip’ that can efficiently predict how a patient will respond to drugs and the outcome of that therapy.

Our last review is *Microfluidics, 3D bioprinted scaffold and organ-on-chip with biosensing activated therapeutic strategies for diabetic wound healing* (Jha et al.).

It brings us readers closer to the organ-on-a-chip devices, which are designed for fighting diabetic foot ulcers (DFUs). The authors of this overview conclude that although there are challenges (e.g., scaling up and standardization), biosensing platforms that are introduced here are promising tools to support more accurate preclinical screening of this disease. These approaches require less labor (and related needs) but at the same time offer non-invasive, multiparametric insight into organ physiology. Organ-on-chip models recapitulate the hyperglycaemic, hypoxic microenvironment of diabetic skin and may improve preclinical screening of DFU-targeted therapeutics. These novel model systems improve the fidelity of *in vitro* and *in vivo* wound models and mimick (simulate) the dynamic conditions of native skin repair well.

Last but not least, I emphasize my gratitude to all of the authors for the manuscripts they have submitted to this Research Topic. I also thank to the Editorial Board for granting the opportunity for these articles to be published here for our readers.

